# Association between oral contraceptives and cervical cancer: A retrospective case–control study based on the National Health and Nutrition Examination Survey

**DOI:** 10.3389/fphar.2024.1400667

**Published:** 2024-07-17

**Authors:** Chong Guo, Bo Zhan, Meng-Yuan Li, Li Yue, Chao Zhang

**Affiliations:** ^1^ Department of Obstetrics and Gynecology, Taihe Hospital, Hubei University of Medicine, Shiyan, Hubei, China; ^2^ Taihe Hospital, Hubei University of Medicine, Shiyan, Hubei, China; ^3^ Center for Evidence-Based Medicine and Clinical Research, Taihe Hospital, Hubei University of Medicine, Shiyan, Hubei, China

**Keywords:** cervical cancer, oral contraceptives, sexual partners, age at first sex, human papillomavirus, National Health and Nutrition Examination Survey

## Abstract

**Background:** Cervical cancer is the fourth most common cancer among females globally, with a high incidence and high mortality among females in developing countries. This retrospective case–control study aimed to investigate the association between oral contraceptives and cervical cancer, on which insufficient evidence still exists.

**Material and Methods:** To examine the association between oral contraceptives and cervical cancer based on 7,496 females aged over 20 years from the National Health and Nutrition Examination Survey, multivariable logistic regression conducted from 1999 to 2016 was used.

**Results:** Contraceptive use was positively associated with cervical cancer risk. In model 1 (unadjusted), a 195% increased risk of cervical cancer was observed among those who used oral contraceptives (odds ratio [OR] = 2.27, 95% confidence interval [CI] = 1.39–3.98, *p* = 0.002) compared to those who did not. In addition, the ORs for the exposed population were 1.74 (95% CI = 1.05–3.08, *p* = 0.041) and 1.93 (95% CI = 1.16–3.44, *p* = 0.017) in model 2 (adjusted for age, race, and body mass index [BMI]) and model 3 (adjusted for education level, ratio of family income to poverty, drinking status, smoking status, number of pregnancies, age at first sex, number of sexual partners, and whether to receive the human papillomavirus (HPV) vaccine in addition to model 2), respectively. Furthermore, subgroup analyses stratified by age, smoking status, BMI, age at first sex, number of sexual partners, and whether to receive the HPV vaccine also revealed that oral contraceptives were significantly associated with cervical cancer.

**Conclusion:** This study demonstrated that oral contraceptive use increased the risk of cervical cancer. In addition, the higher risk, including individuals older than 45 years, having a high BMI (≥30 kg/m^2^), being current smokers, and having more than five sexual partners, may contribute to the development of cervical cancer.

## Introduction

Cervical cancer is the fourth most common cancer among females globally, with a high incidence and high mortality among females in developing countries. The World Health Organization estimates reported 604,000 new cases and 342,000 deaths due to cervical cancer in 2020, and about 90% of the new cases and deaths worldwide in 2020 occurred in developing countries (Sung et al., 2021). Human papillomavirus (HPV) was one of the common risk factors for cervical cancer, and females who were infected with this virus were more likely to develop cervical cancer than those who tested negative. Although there was a consensus that prophylactic HPV vaccination and the availability of Pap smear tests are effective in reducing the incidence of cervical cancer (Van Kriekinge et al., 2014), thousands of individuals continue to develop and die from cervical cancer each year in low- and middle-income countries due to inadequate screening, limited socioeconomic conditions, race, and ethnicity. In addition to HPV, the well-established risk factors, including drinking status, age at first sexual activity, number of sexual partners, smoking status, and body mass index (BMI), were considered risk factors for cervical cancer (Slattery et al., 1989; Agarwal et al., 1993; Castellsagué et al., 2002). A pooled analysis of case–control studies, including 1,864 cases and 1,719 controls, indicated that age at first sex had been associated with an increased risk of cervical carcinoma (Louie et al., 2009). Smoking may increase the risk of cervical cancer by interfering with immunity (Sood, 1991).

Oral contraceptive pills, as one of the most commonly prescribed modern and reversible contraception methods, can effectively control birth for premenopausal females (Mosher and Jones, 2010). However, oral contraceptive use is not without risks. Until recently, the association between oral contraceptives and cervical cancer was not well established. A meta-analysis of case–control studies including 15,619 participants (7,433 cases and 8,186 controls) did not show an association between oral contraceptive use and the risk of cervical cancer (Peng et al., 2017), whereas some studies (Appleby et al., 2007; Roura et al., 2016; Loopik et al., 2020) had reported that the use of oral contraception was one of the risk factors for cervical cancer.

This study investigated the association between cervical cancer and oral contraceptives based on the National Health and Nutrition Examination Survey (NHANES) (Ahluwalia et al., 2016), which is an ongoing series of population-based surveys conducted by the National Center for Health Statistics at the Centers for Disease Control.

## Materials and methods

### Participant selection

**FIGURE 1:**
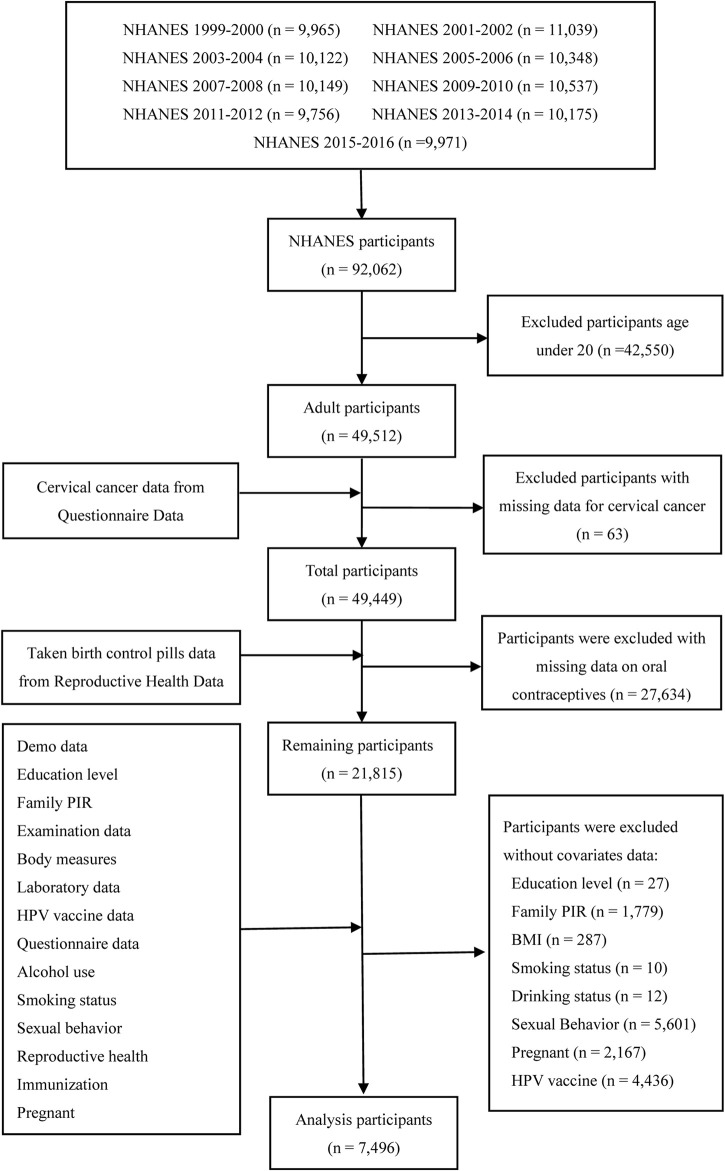
Flowchart for the selection of study participants.

All the data for this study were downloaded from the NHANES program. The gross population sample for this study, comprising 92,062 participants, was selected between 1999 and 2016. Females aged under 20 years (n = 42,550), without cervical cancer data (n = 63), individuals with missing data on oral contraceptive use (n = 27,634), and without covariate data (education level [n = 27], ratio of family income to poverty [family PIR] [n = 1,779], BMI [n = 287], smoking status [n = 10], drinking status [n = 12], sexual behavior [n = 5,601], pregnant [n = 2,167], and HPV [n = 4,436]), were excluded. Finally, a total of 7,496 females were included in this analysis. Figure 1 shows the data of this study used to screen for participants through inclusion and exclusion processes.

### Demographics and covariates

Sociodemographic variables included age, race (Hispanic, non-Hispanic Black, non-Hispanic White, and other races), educational level (college or above, high school grad/GED or equivalent, and less than high school), ratio of family income to poverty (≤1, 1–2, 2–4, and >4), and BMI. The BMI was calculated as BMI = weight (kg)/height (m^2^). According to the criteria of the WHO, BMI <25 kg/m^2^ is normal, BMI = 25.0–29.9 kg/m^2^ is overweight, and BMI ≥30.0 kg/m^2^ is obese. The smoking status was classified as never smoked, former smoker, or current smoker. The drinking status according to the questionnaire was divided into drinking and not drinking. The age at first sex, number of sexual partners, and number of pregnancies were also collected through questionnaires. In addition, whether the HPV vaccine was administered was also obtained using a questionnaire (have you ever received one or more doses of the HPV vaccine?). Information regarding whether patients had cervical cancer was obtained by asking the participants the following problem determinations: 1) Have you ever been told by a doctor or other health professionals that you had cancer or a malignancy of any kind? 2) What kind of cancer was it? Cervical cancer was confirmed if the participant answered yes to the first question and answered cervical cancer to the second question. The above information was obtained through the NHANES questionnaire form for staff, and all the participants provided written informed consent to participate in the investigation of the condition.

### Statistical analysis

Categorical variables were presented as percentages, and continuous variables were presented as means with standard deviation in baseline characteristics. The chi-squared test and the *t*-test were used for categorical variables and continuous variables, respectively. A multivariate logistic regression model was used to examine the association between contraceptive use and cervical cancer. Then, the multivariable logistic regression was calculated for the odds ratio (OR) with 95% confidential interval (CI) of cervical cancer by taking into account oral contraceptive use, as well as stratified by age (Bønløkke et al., 2024), drinking status (Weiderpass et al., 2001), age at first sex (Louie et al., 2009), number of sexual partners (Liu et al., 2015), smoking status (Roura et al., 2014), BMI (Poorolajal and Jenabi, 2016), and whether to receive the HPV vaccine (Walboomers et al., 1999). Taking into account potential confounding factors, three models, model 1 (non-adjusted model), model 2 (adjusted for age, race, and BMI), and model 3 (adjusted for all other co-variables), were used for controlling for the role of covariates. In addition, we stratified the population and used this model to determine whether each factor played a role (promoting, inhibiting, or not) in the association between oral contraceptives and cervical cancer. R 4.2.2 software (R Foundation for Statistical Computing, Vienna, Austria) was used for all statistical analyses, and *p* < 0.05 was considered statistically significant.

## Results

### Characteristics of the study population

The demographic characteristics of participants are shown in Table 1. Of the 7,496 study participants, participants without cervical cancer and with cervical cancer were 7,351 and 145, respectively. The mean age of non-cervical cancer females was 40.64 years, whereas the mean age of cervical cancer patients was 41.56 years. Regarding the race of the females without cervical cancer, 1,334 (18.15%) were Hispanic, 1,703 (23.17%) were non-Hispanic Black, 3,050 (41.49%) were non-Hispanic White, and 1,264 (17.19%) were of other races. Of the females with cervical cancer, 10 (6.90%) were Hispanic, 12 (8.28%) were non-Hispanic Black, 106 (73.10%) were non-Hispanic White, and 17 (11.72%) were of other races. Regarding the educational level of non-cervical cancer females, 4,184 (56.92%) had a college education or above, 2,694 (36.65%) had a high school grad/GED or equivalent education, and 473 (6.43%) had a less than high school education. In cervical cancer females, 68 (46.90%) had a college education or above, 70 (48.28%) had a high school grad/GED or equivalent education, and 7 (4.82%) had a less than high school education. Regarding the BMI in the non-cervical cancer group, 2,156 (29.33%) had BMI <25 kg/m^2^, 3,145 (42.78%) had BMI 25–30 kg/m^2^, and 2,050 (27.89%) had BMI ≥30 kg/m^2^. For the BMI in the cervical cancer group, 40 (27.58%) had BMI <25 kg/m^2^, 68 (46.90%) had BMI 25–30 kg/m^2^, and 37 (25.52%) had BMI ≥30 kg/m^2^. There was a significant difference in the proportion of family PIR groups in the non-cervical cancer population, whereas the cervical cancer population mainly came from a family PIR of less than or equal to 1 (38.62%). Regarding the smoking status, the proportion of current smokers was the highest (53.11%) in the cervical cancer group. Regarding the drinking status, the proportion of drinkers was the highest (71.03%) in the females with cervical cancer. Among cervical cancer patients, contraceptive use and HPV vaccination accounted for 88.97% and 59.31%, respectively. The average age at first sex, the average number of sexual partners, and number of pregnancies in the cervical cancer group were 16.10 years, 12.72, and 3.37, respectively.

**TABLE 1:** Baseline characteristics of participants from nine continuous NHANES datasets.

Characteristic	Without cervical cancer (n = 7,351)	Cervical cancer (n = 145)	*p*-value
Taking oral contraceptive, n (%)			0.0022
Yes	5,734 (78.00)	129 (88.97)	
No	1,617 (22.00)	16 (11.03)	
Age	40.64 (10.68)	41.56 (10.45)	0.3036
Race			<0.0001
Hispanic	1,334 (18.15)	10 (6.90)	
Non-Hispanic Black	1,703 (23.17)	12 (8.28)	
Non-Hispanic White	3,050 (41.49)	106 (73.10)	
Other race	1,264 (17.19)	17 (11.72)	
Education level, n (%)			0.0157
College or above	4,184 (56.92)	68 (46.90)	
High school grad/GED or equivalent	2,694 (36.65)	70 (48.28)	
Less than high school	473 (6.43)	7 (4.82)	
Body mass index, n (%)			0.6078
<25	2,156 (29.33)	40 (27.58)	
25–30	3,145 (42.78)	68 (46.90)	
≥30	2,050 (27.89)	37 (25.52)	
Ratio of family income to poverty, n (%)			0.0001
≤1	1,761 (23.96)	56 (38.62)	
1–2	1,874 (25.49)	34 (23.45)	
2–4	1,871 (25.45)	36 (24.83)	
>4	1,845 (25.10)	19 (13.10)	
Smoking status, n (%)			<0.0001
Current	1,660 (22.58)	77 (53.10)	
Former	1,238 (16.84)	31 (21.38)	
Never	4,453 (60.58)	37 (25.52)	
Drink, n (%)			0.2321
Yes	4,847 (65.94)	103 (71.03)	
No	2,504 (34.06)	42 (28.97)	
Age at first sex, n (%)	17.42 (3.55)	16.10 (3.52)	<0.0001
Sexual partners, n (%)	7.96 (11.64)	12.72 (16.38)	<0.0001
Number of pregnancies, n (%)	3.11 (1.95)	3.37 (1.62)	0.1049
Human papillomavirus, n (%)			0.0003
Positive	3,232 (43.97)	86 (59.31)	
Negative	4,119 (56.03)	59 (40.69)	

Note: *p* < 0.05 indicated statistical significance.

### Association between cervical cancer and oral contraceptives

Table 2 shows the association between cervical cancer and oral contraceptive use based on three models: model 1 (adjusted), model 2 (age, race, and BMI), and model 3 (education level, family PIR, drinking and smoking status, number of pregnancies, age at first sex, number of sexual partners, and HPV vaccination in addition to those included in model 2). Compared with non-oral contraceptives, the use of oral contraceptives significantly increased the risk of cervical cancer in model 1 (OR = 2.27, 95% CI = 1.39–3.98, *p* = 0.002), model 2 (OR = 1.74, 95% CI = 1.05–3.08, *p* = 0.041), and model 3 (OR = 1.93, 95% CI = 1.16–3.44, *p* = 0.017).

**TABLE 2:** Associations between taking oral contraceptive and cervical cancer.

Model	Taking oral contraceptive		*p*-value
	Yes	No	
Model 1	2.27 (1.39, 3.98)	Reference	0.002
Model 2	1.74 (1.05, 3.08)	Reference	0.041
Model 3	1.93 (1.16, 3.44)	Reference	0.017

Note: model 1: unadjusted; model 2: adjusted for age, race, and BMI; model 3: adjusted for education level, family PIR, drinking status, smoking status, number of pregnancies, age at first sex, number of sexual partners, and HPV vaccination, in addition to those included in model 2. *p* < 0.05 indicated statistical significance.

### Stratification analysis

Conducted stratified analyses were further conducted in this study to explore the impact of various factors on the association between oral contraceptive use and cervical cancer risk. The results demonstrated a significant influence of age, BMI, smoking status, age at first sex, number of sexual partners, and HPV vaccination. Women aged 45 years or older, having a high BMI (≥30 kg/m^2^), being current smokers, who had first sex when they were older than 16 years, having more than five sexual partners, and who were HPV vaccinated exhibited an increased risk of cervical cancer with oral contraceptive use.

### Stratification analysis by age

**FIGURE 2:**
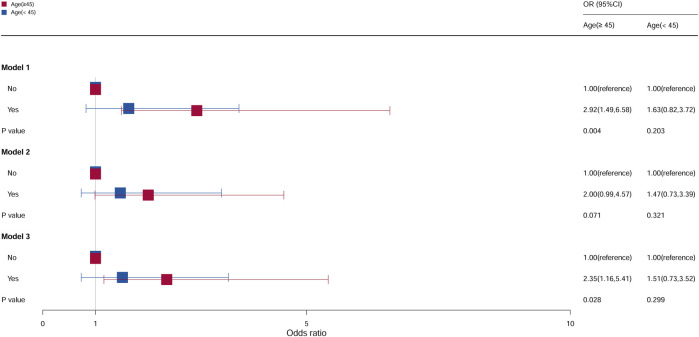
Associations between oral contraceptive use and cervical cancer stratified by age. CI, confidence interval; OR, odds ratio. Model 1: unadjusted; model 2: adjusted for race and BMI; model 3: adjusted for education level, family PIR, smoking status, drinking status, number of pregnancies, age at first sex, number of sexual partners, and HPV vaccination, in addition to those included in model 2. *p* < 0.05 indicated statistical significance.

In subgroup analyses stratified according to age, the population was divided into two groups by an age cutoff of 45 years (Figure 2). Compared with females aged ≥45 years who do not take oral contraceptives, the OR of females who take oral contraceptives was 2.92 (95% CI = 1.49–6.58, *p* = 0.004) in model 1 and 2.35 (95% CI = 1.16–5.41, *p* = 0.028) in model 3, and it was statistically significant. However, for age <45 years, all models were not statistically significant.

### Stratification analysis by BMI

**FIGURE 3:**
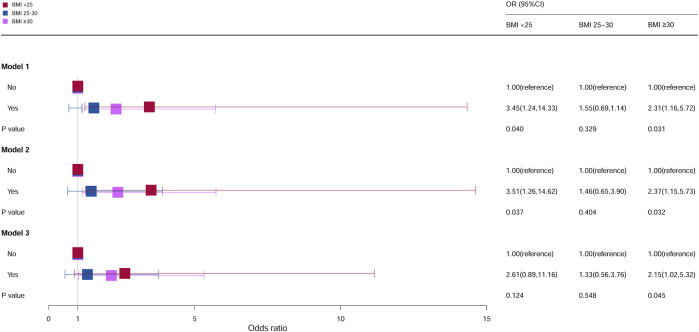
Associations between oral contraceptive use and cervical cancer stratified by BMI. CI, confidence interval; OR, odds ratio. Model 1: unadjusted; model 2: adjusted for age and race; model 3: adjusted for education level, family PIR, smoking status, drinking status, number of pregnancies, age at first sex, number of sexual partners, and HPV vaccination, in addition to those included in model 2. *p* < 0.05 indicated statistical significance.

As shown in Figure 3, compared with females who do not take oral contraceptives with a BMI <25 kg/m^2^, the OR of females who take oral contraceptives was 3.45 (95% CI = 1.24–14.33, *p* = 0.040) in model 1 and 3.51 (95% CI = 1.26–14.62, *p* = 0.037) in model 2, and it was statistically significant. However, it was not statistically significant in model 3. Compared with females who do not take oral contraceptives with a BMI ≥30 kg/m^2^, the ORs of females who took oral contraceptives in model 1, model 2, and model 3 were 2.31 (95% CI = 1.16–5.72, *p* = 0.031), 2.37 (95% CI = 1.15–5.73, *p* = 0.032), and 2.15 (95% CI = 1.02–5.32, *p* = 0.045), respectively. No statistical significance was observed in all models of BMI 25–30 kg/m^2^.

### Stratification analysis by smoking status

**FIGURE 4:**
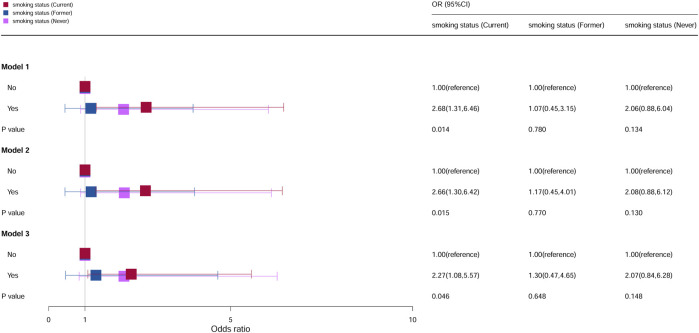
Associations between oral contraceptive use and cervical cancer stratified by smoking. CI, confidence interval; OR, odds ratio. Model 1: unadjusted; model 2: adjusted for age, race, and BMI; model 3: adjusted for education level, family PIR, drinking status, number of pregnancies, age at first sex, number of sexual partners, and HPV vaccination, in addition to those included in model 2. *p* < 0.05 indicated statistical significance.

The association between cervical cancer and oral contraceptive use stratified by smoking is shown in Figure 4. For current smokers, compared with the individuals who do not take oral contraceptives, the risk of cervical cancer increased by 168% among the individuals in model 1 (OR = 2.68, 95% CI = 1.31–6.46, *p* = 0.014), 166% among the individuals in model 2 (OR = 2.66, 95% CI = 1.30–6.42, *p* = 0.015), and 127% among the individuals in model 3 (OR = 2.27, 95% CI = 1.08–5.57, *p* = 0.046), and it was statistically significant. However, for former smokers and individuals who never smoked, all models were not statistically significant.

### Stratification analysis by age at first sex

**FIGURE 5:**
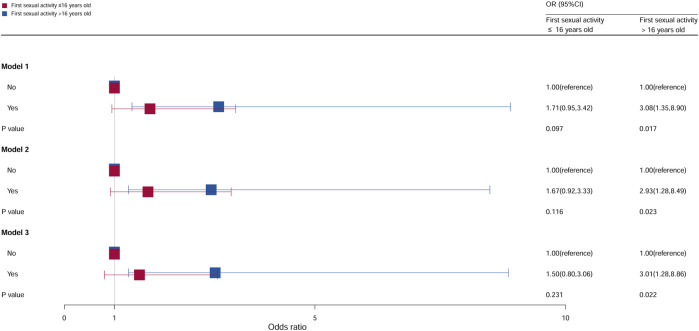
Associations between oral contraceptive use and cervical cancer stratified by age at first sex. CI, confidence interval; OR, odds ratio. Model 1: unadjusted; model 2: adjusted for age, race, and BMI; model 3: adjusted for education level, family PIR, smoking status, drinking status, number of pregnancies, number of sexual partners, and HPV vaccination, in addition to those included in model 2. *p* < 0.05 indicated statistical significance.

The population was divided into two groups based on the age at first sex (first sexual activity at ≤16 years and first sexual activity at >16 years). The relation between age at first sex and cervical cancer was significant, as shown in Figure 5. Compared with females who did not take oral contraceptives in first sexual activity at >16 years, the OR of females who take oral contraceptives was 3.08 (95% CI = 1.35–8.90, *p* = 0.017) in model 1, 2.93 (95% CI = 1.28–8.49, *p* = 0.023) in model 2, and 3.01 (95% CI = 1.28–8.86, *p* = 0.022) in model 3. However, no statistical significance was observed in all models of first sex at >16 years.

### Stratification analysis by number of sexual partners

**FIGURE 6:**
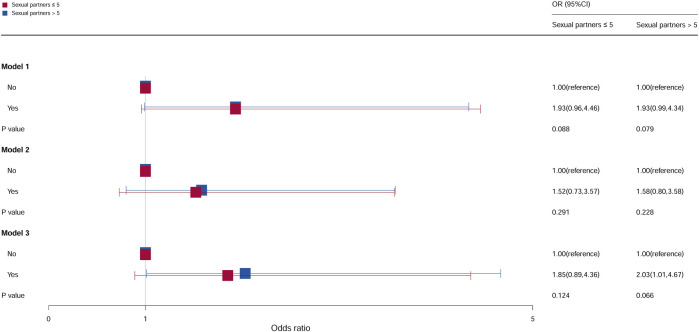
Associations between oral contraceptive use and cervical cancer stratified by the number of sex partners. CI, confidence interval; OR, odds ratio. Model 1: unadjusted; model 2: adjusted for age, race, and BMI; model 3: adjusted for education level, family PIR, smoking status, drinking status, number of pregnancies, age at first sex, and HPV vaccination, in addition to those included in model 2. *p* < 0.05 indicated statistical significance.

Multivariate analyses stratified by the number of sexual partners showed that the number of sexual partners was significantly related to cervical cancer incidence, as shown in Figure 6. Compared with females who do not take oral contraceptives when the number of sexual partners >5, the ORs of females who take oral contraceptives were 2.03 (95% CI = 1.01–4.67, *p* = 0.066) in model 3, and no statistical significance was observed in the other models. For females with sexual partners ≤5, no statistical significance was found.

### Stratification analysis by whether received the HPV vaccine

**FIGURE 7:**
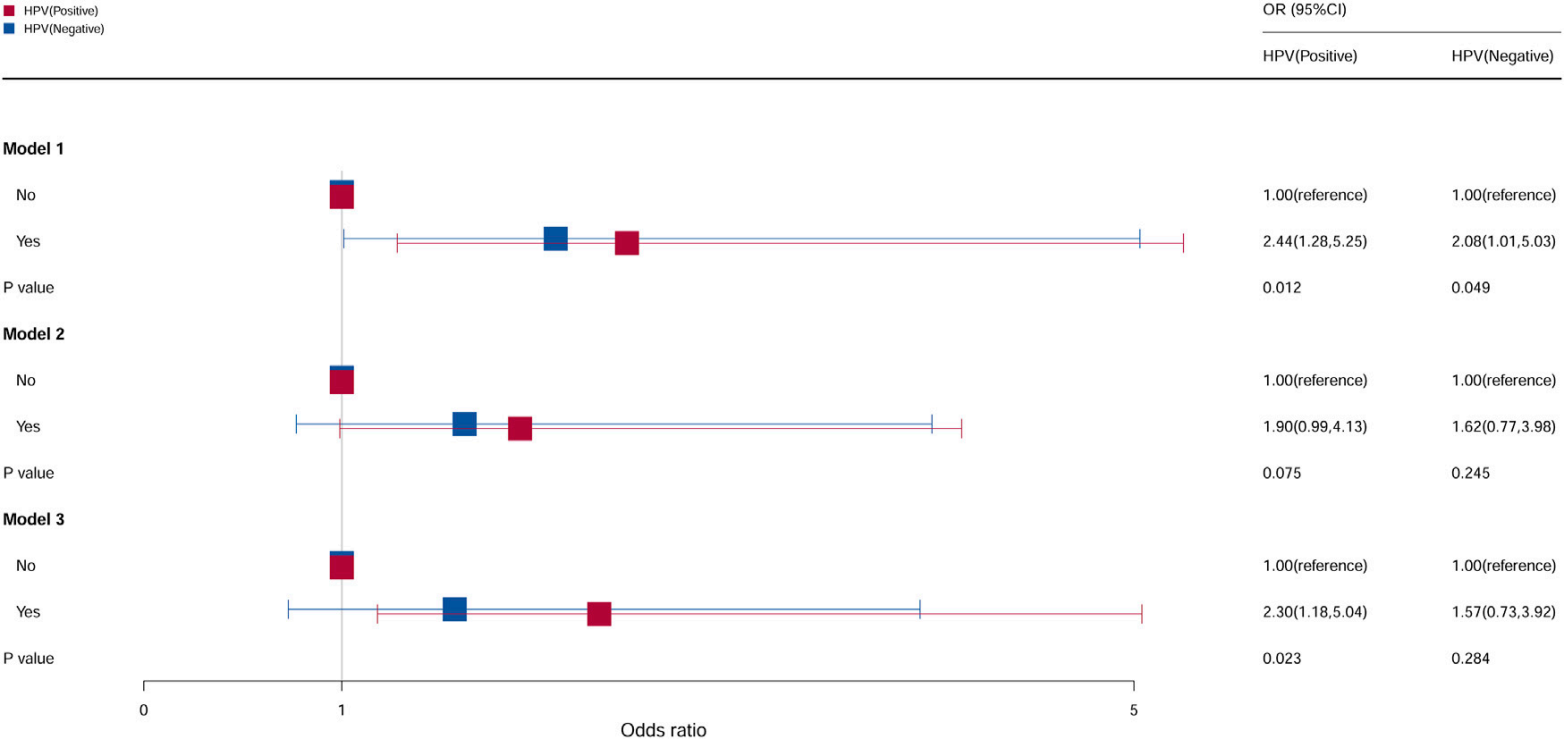
Associations between oral contraceptive use and cervical cancer stratified by HPV vaccination. CI, confidence interval; OR, odds ratio. Model 1: unadjusted; model 2: adjusted for age, race, and BMI; model 3: adjusted for education level, family PIR, drinking status, smoking status, number of pregnancies, age at first sex, and number of sexual partners, in addition to those included in model 2. *p* < 0.05 indicated statistical significance.

Compared with females who do not take oral contraceptives, as shown in Figure 7, the risk of cervical cancer for the HPV vaccination population who used contraceptive pills was 2.44, and it was 2.30 times higher in models 1 and 3 (model 1: OR = 2.44, 95% CI = 1.28–5.25, *p* = 0.012; model 3: OR = 2.30, 95% CI = 1.18–5.04, *p* = 0.023). Compared with females who do not take oral contraceptives in the non-HPV vaccination population, the OR of females who take oral contraceptives was 2.08 (95% CI = 1.01–5.03, *p* = 0.049) in model 1. No statistical significance was found in all the other groups.

## Discussion

In the study, multivariable logistic regression proved that taking oral contraceptives increased the risk of cervical cancer. The use of the pill remained strongly associated with cervical cancer after controlling for covariates, including age, race, BMI, education level, family PIR, drinking status, smoking status, number of pregnancies, age at first sex, number of sexual partners, and whether to receive the HPV vaccine. Further stratification analysis showed that taking oral contraceptives was strongly associated with cervical cancer.

Oral contraceptives are currently one of the most common forms of contraception, which are common among many young people (Cromwell and Daley, 2000; Daniels et al., 2014). Oral contraceptives are mainly divided into progestin-based contraceptives and combined oral contraceptives. The main principles of these drugs are to inhibit ovulation, hinder the combination of sperm and eggs, and interfere with the implantation of fertilized eggs to achieve the purpose of contraception. However, contraceptives may be a risk factor for a number of diseases, including cervical cancer. An epidemiological study including 16,573 females with cervical cancer and 35,509 without cervical cancer showed that the long-term contraceptive use was a clear risk factor for cervical cancer (Appleby et al., 2007). Several systematic reviews also supported the conclusions of this study (Delgado-Rodriguez et al., 1992; Smith et al., 2003; Asthana et al., 2020). However, the exact mechanisms linking oral contraceptives and cervical cancer are less clear.

It was well known that age is an important risk factor for cervical cancer (Bønløkke et al., 2024). In subgroup analyses stratified by age, the risk for cervical cancer varied by age among oral contraceptive users. Compared with young women, older women taking oral contraceptives are more likely to develop cervical cancer. With the availability of the HPV vaccine, the number of women with cervical cancer has decreased in recent years. However, in developing countries or regions, the number of cervical cancer patients is still high. Therefore, the effect of age may be more pronounced in these countries or regions. In this study, women were recommended to avoid the long-term use of oral contraceptives, and HPV vaccination was recommended as early as possible at an appropriate age. In addition, the awareness of cervical cancer still has a long way to go, and the overall level of global medical development needs to be improved.

BMI was included as a risk factor for cervical cancer due to increasing evidence that obesity is associated with various cancers. A meta-analysis by Poorolajal and Jenabi (2016) demonstrated that being overweight is not associated with an increased risk of cervical cancer, but obesity is weakly associated with an increased risk of cervical cancer. After the adjustment of all variables, this study also found that being overweight (BMI ≥30 kg/m^2^) was not associated with the incidence of cervical cancer. However, it is worth noting that Urbute et al. (2024) reported that a higher-than-normal BMI was associated with higher incidence rates of cervical cancer and lower rates of pre-cancer detection. Relevant research studies (Papadia et al., 2006; Aldrich and Hackley, 2010; Sand et al., 2023) suggested that the higher risk of cervical cancer among obese females may be due to a lower adherence to cervical cancer screening among them. In addition, Clark et al. (2016) showed that both extremes of weight (underweight and overweight/obesity) were associated with worse survival in patients with cervical cancer. Optimizing the weight in cervical cancer patients may improve their outcomes. However, the confidence intervals of underweight and overweight females based on the results of this research were wide, resulting in the low precision and accuracy of the results. Therefore, the conclusions for this population still need to be verified by larger samples.

The role of smoking in the risk of cancer, including cervical cancer, had long been studied by research workers (Mzarico et al., 2015). In recent years, with the increasing number of female smokers, smoking as one of the risk factors of cervical cancer (Roura et al., 2014) was further studied. Some studies (Pate et al., 2009; Su et al., 2018; Kim et al., 2021) showed that passive smoking also increased the risk of cervical cancer in females. In this study, the effect of current smoking on contraceptive use and cervical cancer can increase the risk of cervical cancer. Smoking increased the risk of cervical cancer by two times compared to non-smokers (Plummer et al., 2003; Vaccarella et al., 2008). The specific mechanism between smoking and cervical cancer was not well established, but the impaired immune response caused by smoking and the different levels of oxidative DNA damage induced by free radicals provided a biological plausibility for the link between tobacco and cervical cancer (Eiserich et al., 1995; Poppe et al., 1995; Mena et al., 2009). Therefore, this study suggested that smoking should be controlled and reduced to reduce the risk of cervical cancer.

In this study, both age at first sex and number of sexual partners were associated with cervical cancer risk. In a comparative study based on US and Italian populations, cervical cancer risk was increased with multiple partners, younger age at first sex, and use of oral contraceptives (Parazzini et al., 1990). The association between age at first sex and number of sexual partners and cervical cancer may be mediated through HPV. Younger age at first sex and more sexual partners were associated with a higher likelihood of HPV infection (Gravitt and Jamshidi, 2005; Remschmidt et al., 2014). In our study, the risk of cervical cancer among women who had first sexual intercourse after the age of 16 years was higher among those who had used oral contraceptives than among those who had not. However, when the first sexual intercourse is when the female was younger than 16 years, oral contraceptives have no effect on the occurrence and development of cervical cancer. This may be due to the small number of data on cervical cancer in this study. Therefore, these results still need to be verified, and evidence reconstruction should be carried out using large samples.

Related studies demonstrated that HPV infection can increase the risk of cervical cancer (Zhang et al., 2020). Hildesheim et al. (1990) showed a 2.3-fold and 2.9-fold increased risk of HPV positivity among females with recent or long-term (>4 years) use of oral contraceptives, respectively, suggesting the increased expression of the HPV genome in tumors of oral contraceptive users. It is well known that HPV infection alone was considered necessary but not sufficient to develop cervical cancer, and HPV may also interact with other factors such as smoking (Hildesheim et al., 1990; Castle et al., 2002). Therefore, HPV vaccination can reduce the incidence of cervical cancer (Falcaro et al., 2024), and the elimination of cervical cancer can be approached through increased HPV vaccination rates and efforts to increase cervical cancer screening, particularly screening to close disparities around the world (Rahangdale et al., 2022).

This study supported a multi-factorial model of cervical cancer etiology. Therefore, this study suggested that health education should be strengthened to improve the health awareness and compliance of females to cervical cancer screening, so as to reduce the risk of cervical cancer in females.

## Limitations

There were several limitations to this study. First, the included sample in this study was small, especially the group of cervical cancer females, and our results need to be verified with a larger sample size. Second, a retrospective case–control study cannot establish causality, and this study may have recall bias. Third, different oral contraceptives, dosage, HPV infection, family history of genetics, and so on affected the risk of cervical cancer. Fourth, in cohort studies, the measurement of exposure typically occurs at the baseline. If the exposure status of participants changes after the baseline, these alterations may not be captured, thereby affecting the interpretation of the results. In addition, the inclusion and exclusion criteria limited the sample size, such as cases aged between 20 and 60 years, which also limited the population to which this evidence can be extended. The conclusion that oral contraceptive use was more likely to develop cervical cancer in HPV-vaccinated population is probably due to the fact that the data on cervical cancer involved in this study are too small, and the results need to be evaluated and verified by larger samples and more comprehensive information.

## Conclusion

In summary, this study demonstrated that oral contraceptive use increased the risk of cervical cancer. In addition, the higher risk, including in individuals older than 45 years, having a high BMI (≥30 kg/m^2^), being current smokers, and having more than five sexual partners, may contribute to the development of cervical cancer. More studies are needed to further validate the association between oral contraceptives and cervical cancer and to explore the underlying mechanisms.

## Data Availability

Publicly available datasets were analyzed in this study. All data in the study are available at https://www.cdc.gov/nchs/nhanes/index.htm.
